# Population health and sociodemographic variables as predictors of access to cardiac medicine and surgery in Haiti

**DOI:** 10.1186/s41256-023-00308-z

**Published:** 2023-07-19

**Authors:** Esha Bansal, Krishna Patel, Samantha Lacossade, Bennisoit Gue, Kessy Acceme, Owen Robinson, Gene F. Kwan, James R. Wilentz

**Affiliations:** 1 Arnhold Institute of Global Health, Icahn School of Medicine at Mount Sinai, 1 Gustave L Levy Place, New York, NY 10029 USA; 2 Saint Damien Pediatric Hospital, Nos Petits Frères et Sœurs, Port-au-Prince, Haiti; 3 Haiti Cardiac Alliance, 47 Maple Street, Suite 213, Burlington, VT 05401 USA; 4 Section of Cardiovascular Medicine, Boston University School of Medicine, 72 East Concord St, Boston, MA 808 USA

**Keywords:** Cardiac care access, Haiti, Geographic disparities, Socioeconomic disparities, Health equity, Global surgery, Congenital heart disease, Rheumatic heart disease

## Abstract

**Background:**

In Haiti, cardiovascular disease is a leading cause of morbidity and mortality, with congenital and rheumatic heart disease comprising a large portion of disease burden. However, domestic disparities in cardiac care access and their impact on clinical outcomes remain poorly understood. We analyzed population-level sociodemographic variables to predict cardiac care outcomes across the 10 Haitian administrative departments.

**Methods:**

This cross-sectional study combined data from a 2016–17 Haitian national survey with aggregate outcomes from the Haiti Cardiac Alliance (HCA) database (n = 1817 patients). Using univariate and multivariable regression analyses, the proportion of HCA patients belonging to each of three clinical categories (active treatment, lost to follow-up, deceased preoperatively) was modeled in relation to six population-level variables selected from national survey data at the level of the administrative department.

**Results:**

In univariate analysis, higher department rates of childhood growth retardation were associated with a lower proportion of patients in active care (OR = 0.979 [0.969, 0.989], *p* = 0.002) and a higher proportion of patients lost to follow-up (OR = 1.016 [1.006, 1.026], *p* = 0.009). In multivariable analysis, the proportion of department patients in active care was inversely associated with qualified prenatal care (OR = 0.980 [0.971, 0.989], *p* = 0.005), and child growth retardation (OR = 0.977 [0.972, 0.983]), *p* = 0.00019). Similar multivariable results were obtained for department rates of loss to follow-up (child growth retardation: OR = 1.018 [1.011, 1.025], *p* = 0.002; time to nearest healthcare facility in an emergency: OR = 1.004 [1.000, 1.008, *p* = 0.065) and for preoperative mortality (prenatal care: OR = 0.989 [0.981, 0.997], *p* = 0.037; economic index: OR = 0.996 [0.995, 0.998], *p* = 0.007; time to nearest healthcare facility in an emergency: OR = 0.992 [0.988, 0.996], *p* = 0.0046).

**Conclusions:**

Population-level survey data on multiple variables predicted domestic disparities in HCA clinical outcomes by region. These findings may help to identify underserved areas in Haiti, where increased cardiac care resources are required to improve health equity. This approach to analyzing clinical outcomes through the lens of population-level survey data may inform future health policies and interventions designed to increase cardiac care access in Haiti and other low-income countries.

## Background

In recent years, cardiovascular disease (CVD) has become the leading cause of morbidity and mortality in low- and middle- income countries (LMICs). Haiti, where an estimated 29% of population mortality is attributed to cardiovascular etiologies [1], is no exception to this pattern. For instance, an estimated 49% of Haitian women and 38% of Haitian men ages 35–64 years have hypertension [1], while the median age for ischemic and hemorrhagic stroke is 10 years younger than in other LMICs [2].

Cardiovascular pathology in Haiti, however, does not perfectly mirror that of high-income countries, which are predominantly burdened by adult atherosclerotic cardiovascular disease (ASCVD) [3]. Given Haiti’s bottom-heavy population pyramid with nearly one third of inhabitants below the age of 15 [4, 5], cardiac diseases of childhood and young adulthood—including unrepaired congenital heart disease (CHD), rheumatic heart disease (RHD) and nonischemic forms of heart failure like peripartum cardiomyopathy [6, 7]—cause substantially greater morbidity and mortality for Haitians than for the global population as a whole. In 2019, 1.50% of the Haitian population died from complications of CHD and 0.73% of the Haitian population died from RHD, in comparison to the respective death indices of 0.38% and 0.54% among the aggregate world population. Concurrently, RHD generated 0.66% of disability-adjusted life years (DALYs) in Haiti yet is responsible for only 0.42% of total worldwide DALYs, whereas congenital heart anomalies are responsible for 2.15% of Haitian DALYs compared to 0.74% of all DALYs globally [8]. Indeed, Haiti is one of only seven countries with an age-standardized mortality rate for congenital heart disease twice greater than the world average [9] and its peripartum cardiomyopathy rate of 1 per 300 deliveries is believed to be the second-highest in the world [10].

Beyond these epidemiologic complexities, the geospatial and resource limitations of the Haitian CVD landscape necessitate novel, targeted public health approaches. The country’s extreme poverty and minimal infrastructure have severely constrained the availability of basic cardiac screening, diagnostic, and therapeutic modalities [11]. It is critical to note that CHD and RHD frequently require surgical or interventional repair, which is particularly challenging to deliver at scale due to higher costs and resource requirements compared to medical therapy alone. There is only one pediatric cardiologist for four million children in Haiti, and only six general surgical, anesthetic, or obstetric providers per 100,000 people [12]. Nationally, just 33% of Haitian hospitals have blood banks [13] and no minimally-invasive (diagnostic or interventional catheterization laboratory) or open-heart surgical programs exist to date [14]. Since 2021, the acute crisis of political and social instability in Haiti has further exacerbated these shortages by either completely curtailing or sharply circumscribing the operations of many on-the-ground medical aid providers. As a result, a significant number of Haitians currently forgo life-saving medical surveillance and cardiac procedures that are routine and readily available in high-income countries.

For Haitian patients, financial and social barriers to specialty healthcare are similarly life-limiting. 59% of Haitians live under the national poverty line of $2.41 per person per day [15]. Meanwhile, a pediatric cardiac consultation with echocardiogram in Port-au-Prince costs between $20 (non-profit children’s hospital) and $60–90 (private sector) [14]. With user fees present at 93% of health facilities, and given existing trust barriers toward Western medicine, many Haitians seek care from untrained, unregulated healers and are thus more likely to suffer delays in adequate care, reductions in quality of care, and treatment complications including disability and death [16].

Within this complex picture, regional heterogeneities in the Haitian healthcare landscape merit additional consideration. Haiti is divided into 10 administrative departments, and national surveys indicate substantial health disparities between urbanized departments like *West* (where Port-au-Prince, the most populous city of Haiti, is located) and more rural departments such as *Center* and *Northeast* [15]. However, research on healthcare disparities across the different departments of Haiti, and their impact on clinical outcomes, is almost nonexistent in the current academic literature.

At present, clinical interventions and health outcomes research in Haiti are primarily driven by non-profit and non-governmental organizations, and a major scarcity of data limits evidence-based intervention design. In 2020, Yan et al. reported that patients attending an adult primary care clinic in Mirebalais, Haiti had relatively greater economic well-being than the broader Haitian population. Authors suggested that patients with more favorable economic status were better able to overcome significant geographic and travel-related obstacles to care [17]. Beyond Yan et al., the only other cardiovascular health disparities research in Haiti to our knowledge is that of Lookens et al., whose ongoing longitudinal study of 3000 urban adults with CVD evaluates underlying risk factors and social determinants of health [11]. Further research, particularly in relation to Haitian CHD and RHD populations, is largely absent despite the magnitude of unmet health needs. To address this gap in understanding of cardiovascular health equity and, specifically, access to CHD and RHD care in Haiti, this ecological study seeks to analyze department-level outcomes from the largest known Haitian cardiac patient database, and to analyze these outcomes in relation to national survey data on population health and sociodemographic indices. Using these indices as predictors, we aim to understand how clinical outcomes differ across the 10 Haitian administrative departments. In doing so, we attempt to establish an analytic approach that may be generalizable to other forms of specialty healthcare and to other low-income countries.

## Methods

### Study context

**Fig. 1:**
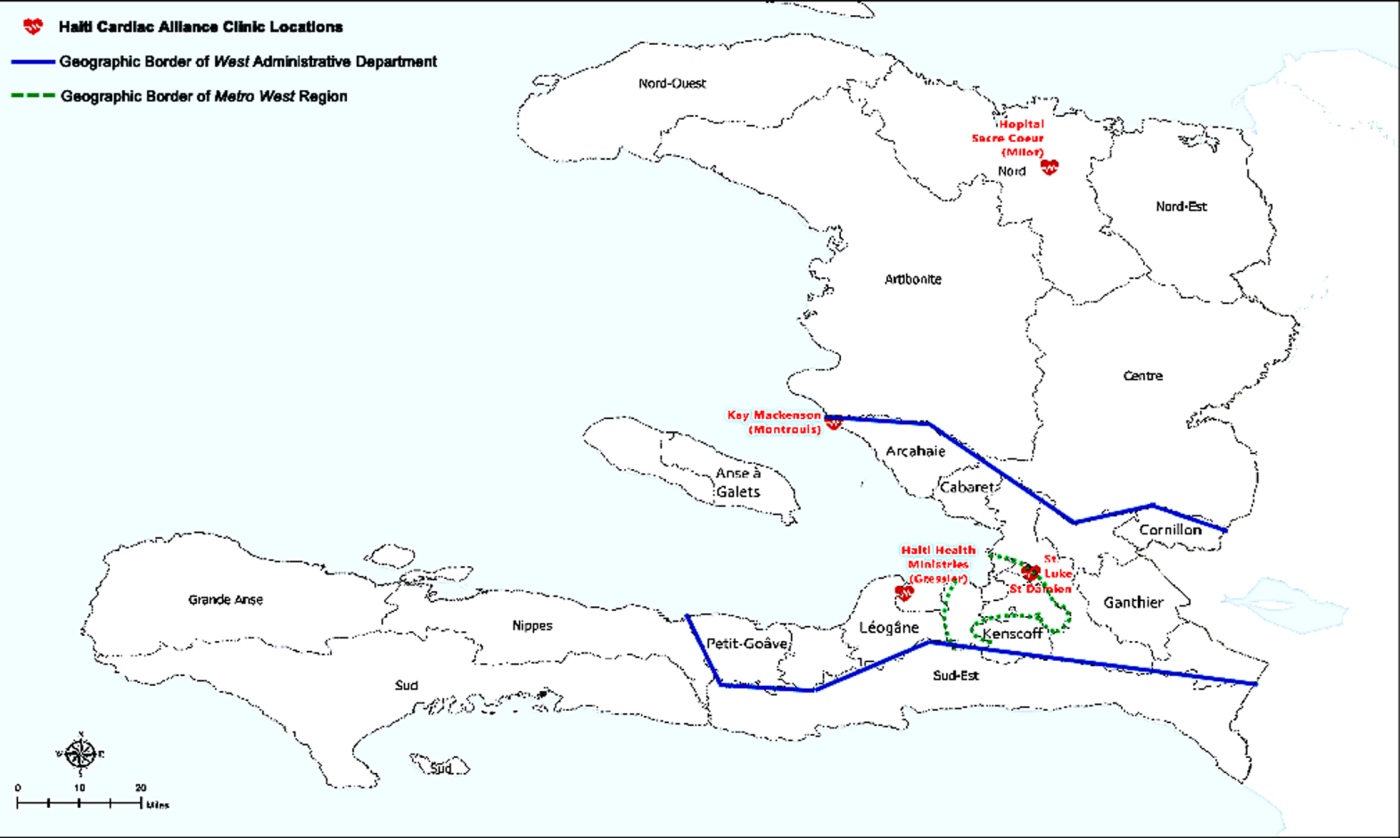
Map of Haiti by administrative department, showing the locations of the five Haiti Cardiac Alliance clinics. Red heart icons indicate the locations of Haiti Cardiac Alliance clinical sites. Blue line denotes the borders of the *West* administrative department; green dashed line marks the periphery of the *Metro West r*egion

Founded in 2013, Haiti Cardiac Alliance (HCA) is a US-based non-governmental organization, with the mission of facilitating access to cardiac diagnosis, surgery, and medical care for Haitian children and young adults with heart disease. In partnership with Haitian medical providers and international surgical centers, HCA has facilitated approximately 500 cardiac procedures and provides long-term cardiac surveillance and medication management for over 1000 Haitian children and young adults annually. HCA and its hospital partners perform cardiac consultations at five clinical sites throughout Haiti: St. Damien, St. Luke, Gressier, Montrouis, and Milot (Fig. 1). Patients enter HCA care via referrals (placed by email or telephone) from local primary care clinics, hospitals, private-sector cardiologists, and social service organizations. All HCA services are free of charge to patients. The most common presenting pathologies of HCA patients are RHD and CHD. Upon establishment of care and at specified intervals thereafter, patients receive a clinical evaluation, echocardiography, treatment initiation, and accompaniment for follow-up medical and surgical care as indicated. For surgical candidates, preoperative consultation typically occurs six months prior to planned surgery and routine follow up occurs at 1, 3, 6, 12, 24, and 60 months postoperatively. Patients have been first seen by HCA at any age ranging from newborn to 38 years.

### Variables and data sources

Dependent variables in this analysis are aggregate statistics derived from an HCA source database. The source database contained de-identified information based on patients (n = 1817) who either received or awaited cardiac care from April 1, 2012 to December 23, 2020. Data regarding patient age, sex, administrative department, and clinical outcome were analyzed. Only patients found to have missing clinical outcome and/or administrative department data were excluded from analysis. Within the study cohort subjected to these inclusion / exclusion criteria, there were no additional missing data items. Of note, prior internal review of HCA data concluded that, during the time course of this observational study, patient movement across administrative departments occurred in fewer than 1% of cases, so this factor was considered negligible for the purposes of data analysis.

The primary dependent variable of interest, clinical outcome, was categorically defined as one of the following: a) active care; b) lost to follow-up; c) deceased preoperatively. *Lost to follow-up* denoted patients unable to be reached on all provided contact numbers for three consecutive months and *deceased preoperatively* represented confirmed deaths prior to indicated surgery. *Active care* referred to all other patients, including surgical candidates in preoperative and postoperative care as well as non-surgical patients receiving medical management and routine follow-up, as defined at the time of analysis. On average, patients in *active care* were seen at least annually for examination and echocardiography. Deaths among postoperative and non-surgical patients who were under active care at the time of death were likewise included in active care. If surgical patients had initially lost contact with HCA and were later discovered to have died preoperatively, they were classified as *lost to follow-up* because their death was attributed to a lack of cardiac care continuity.

Haitian population data by administrative department were extracted from the government-administered *2016–2017 Enquête Mortalité, Morbidité et Utilisation des Services* (EMMUS-VI) *[Study of mortality, morbidity and utilisation of services]*, the most recent dataset of health and economic outcomes across Haiti at the time of this analysis [1]. The extracted independent variables, used to predict HCA departmental clinical outcomes, were as follows (Table 1) [1]:

**Table 1:** Definition of population-level variables utilized in analysis, adapted from the *2016–2017 Enquête Mortalité, Morbidité et Utilisation des Services* (EMMUS-VI)* survey*

Variable	Definition
Access to qualified prenatal care	Percentage of women aged 15–49 years who had a live birth and received prenatal care from a qualified provider (physician, nurse, or midwife)
Early child mortality	Average number of children dying between date of birth and fifth birthday, per 1000 live births
Child growth retardation	Percentage of children under age 5 years with a height-for-age Z score of less than − 2, based on a representative sample
Adult employment	Percentage of adults with active employment in the 7 days prior to survey administration (average of reported male and female statistics)
Economic index	On a scale of 0–100, the average percentile of economic well-being for department households, calculated based on the percent of surveyed households belonging to each of the five national income quintiles (quintile 1: 0–19.9%, quintile 2: 20–39.9%, quintile 3: 40–59.9%, quintile 4: 60–79.9%, quintile 5: 80–100%)
Average travel time to the nearest healthcare facility in an emergency	Average travel time to nearby healthcare facility in an emergency, using any travel means available, among seriously injured or ill members of surveyed households who were brought to a healthcare facility within 30 days before survey administration. This was calculated as a weighted average using mean values of survey-specified intervals (0–15, 15–29, 30–59, 60–119, or 120 + minutes) and excluding those with unknown travel time

Extracted variables were selected a priori on the basis of relevance to previous literature concerning the determinants of health outcomes in LMICs; there was intent to include parameters broadly inclusive of child health (early child mortality, growth retardation), economic well-being (adult employment, economic index), and access to care (access to prenatal care, average travel time) [1, 11, 17–19]. All statistical models were assessed for collinearity among these factors.

In both the HCA and EMMUS-VI datasets, *West*, Haiti’s most populous administrative department, was divided into two subsections: 1) “Metropolitan West,” consisting of the Port-au-Prince, Delmas, Cite Soleil, Tabarre, Carrefour and Pétion-Ville communes; and 2) “Rest of West” containing all other communes. This convention was followed to delineate urban and rural zones of the department, thereby dividing the disproportionately large *West* population accordingly.

### Statistical analysis

The distribution of the HCA patient base across all Haitian administrative departments was calculated from the HCA clinical database and compared with that of the general Haitian population. Descriptive statistics and counts for sex, age, and clinical outcome by department were also computed from the source database. Descriptive statistics encompassed calculation of: the total number of patients and percentage of patients from each administrative department, the sex breakdown of patients by department (reported as percentage of females), the mean patient age and interquartile range of patient age by department, the percentage of HCA patients belonging to each department, and the total number and percentage of patients belonging to each clinical outcome category by department. The quantity of HCA patients belonging to a particular clinical outcome in each department was also visually represented in ArcGISPro 2.9.1 [20], both in terms of absolute statistics and as a proportion of the total HCA clinical database. When using ArcGIS to generate figures, quintiles were applied as legend thresholds because this provided the best overall visualization of interdepartmental differences.

Regression analyses were then performed to evaluate the association of each departmental clinical outcome (active care, lost to follow-up, or deceased preoperatively) with the corresponding department-level health and economic indices from the EMMUS-VI. In univariate models, odds ratios, 95% confidence intervals (CIs), and p-values were calculated for all six EMMUS-VI covariates to evaluate the extent to which each of these was predictive of HCA clinical outcomes. Next, multivariable modeling was performed to assess for independent, department-level associations between each EMMUS-VI covariate and the proportion of HCA patients belonging to the three clinical outcomes, after adjusting for all of the other covariates (prenatal care, early child mortality, child growth retardation, adult employment, economic index, and time to the nearest healthcare facility).

Of note, all multivariable regression models were subject to multiple quality controls. Variance-inflation factor (VIF) calculations were performed to assess collinearity between covariates. Residuals vs. fitted, QQ, scale-location, and Cook’s Distance plots were also performed as additional robustness checks to check for over-fitting. In multivariable models with VIF > 10 for any variable or plots that suggested violation of a fundamental multivariable model assumption, the number of variables was pruned [21]. Multiple R^2^ and F statistics were also calculated for the multivariable models. For all regression analyses, two-sided *p*-values < 0.05 were considered statistically significant in all interpretations of analyses. All statistical analyses were performed in RStudio Version 1.3.959.

## Results

### HCA clinical statistics

**Fig. 2:**
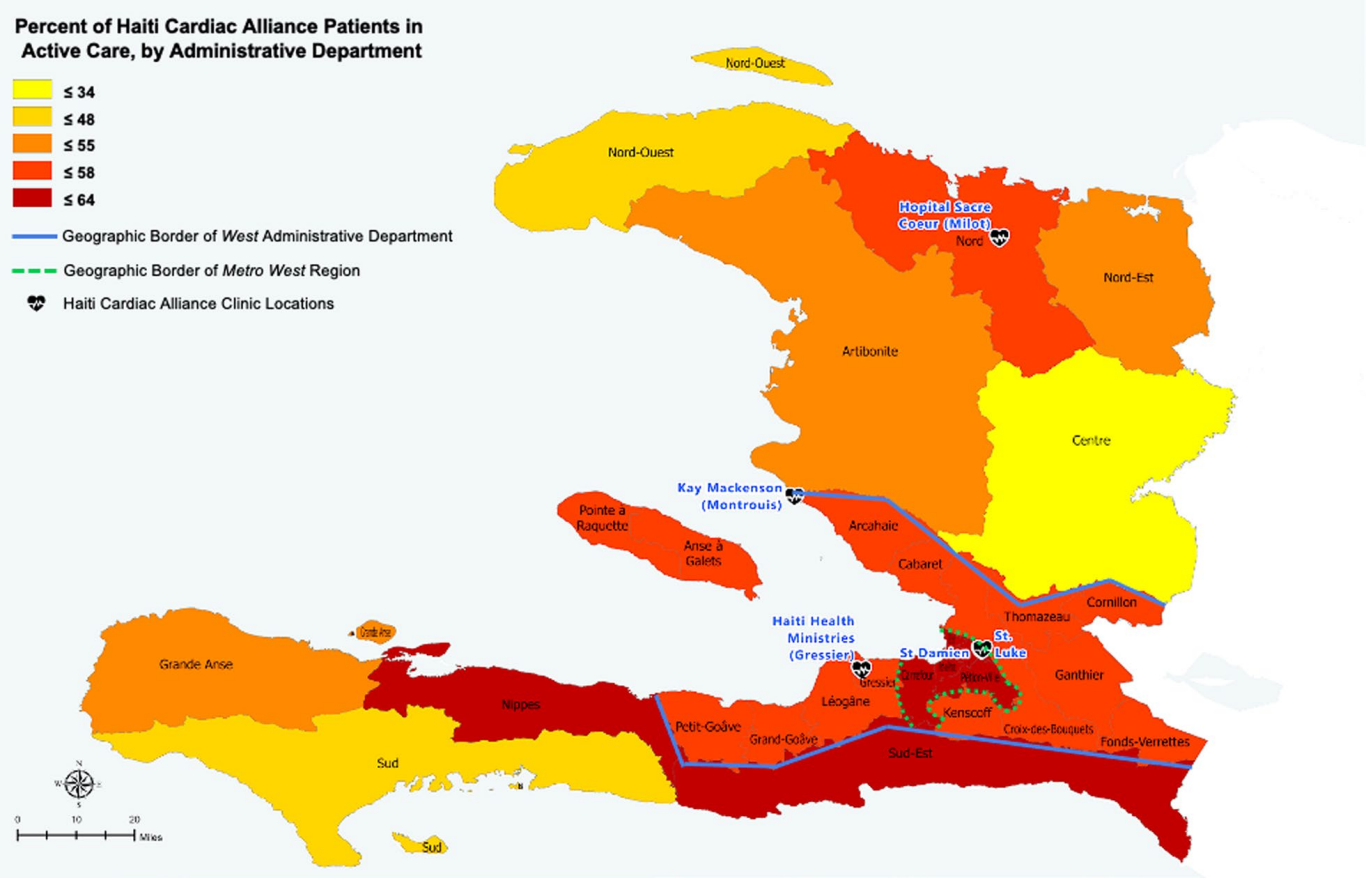
Percent of Haiti Cardiac Alliance patients in active care, by administrative department. Blue line denotes the borders of the West department; green dashed line marks the periphery of Metro West. Black heart icons indicate the locations of Haiti Cardiac Alliance clinics. Legend quintiles were selected to optimize data visualization

**Fig. 3:**
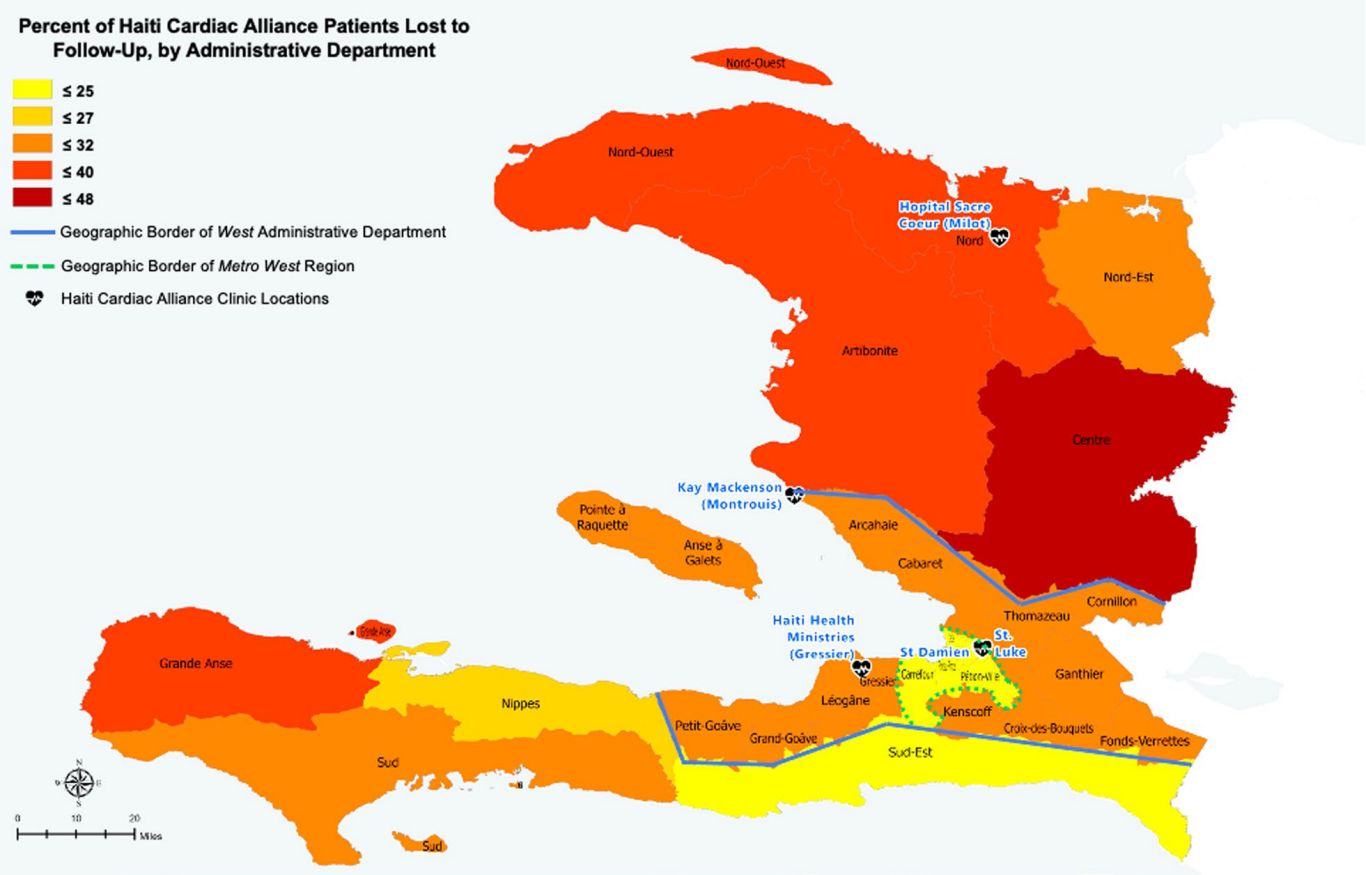
Percent of Haiti Cardiac Alliance patients lost to follow-up, by administrative department. Blue line denotes the borders of the West department; green dashed line marks the periphery of Metro West. Black heart icons indicate the locations of Haiti Cardiac Alliance clinics. Legend quintiles were selected to optimize data visualization

**Fig. 4:**
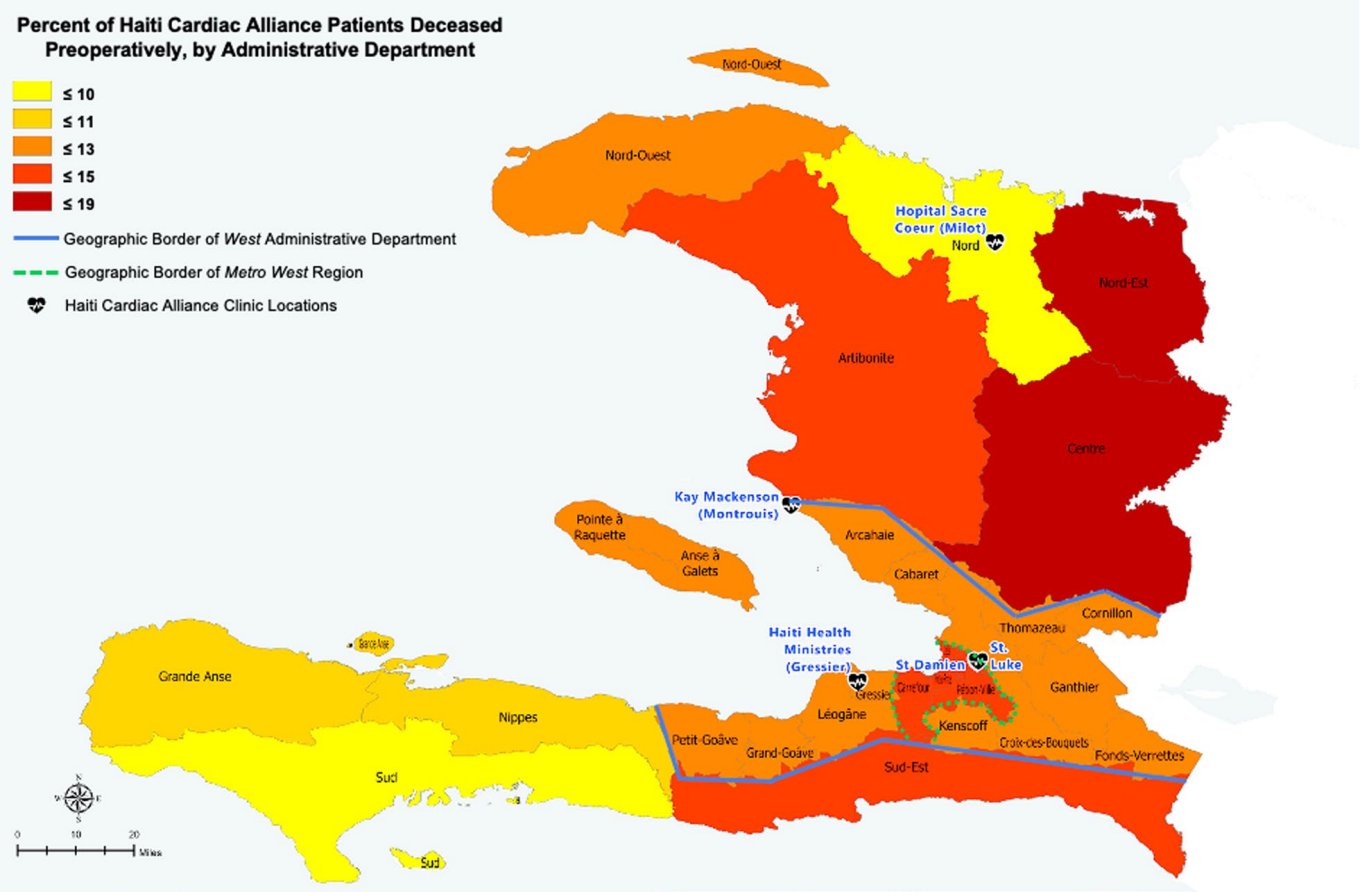
Percent of Haiti Cardiac Alliance patients deceased preoperatively, by administrative department. Blue line denotes the borders of the West department; green dashed line marks the periphery of Metro West. Black heart icons indicate the locations of Haiti Cardiac Alliance clinics. Legend quintiles were selected to optimize data visualization

The overall patient base (n = 1817) was 52% female with a median age of 5.87 years (IQR: 2.16–12.31 years) and resided in all 10 departments, including both the *Metro West* and *Rest of West* sections of the *West* department. In terms of clinical outcome groups, *active care* comprised 57.4% of the database (n = 1043), *lost to follow-up* comprised 29.1% (n = 529), and *deceased preoperatively* comprised 13.5% (n = 245). A detailed breakdown of HCA aggregate statistics by department is included in Table 2, while Figs. 2, 3 and 4 visually depict clinical outcomes at the department level.

**Table 2:** Description of the Haiti Cardiac Alliance study population, by administrative department

	Total (n)	Sex (%F)	Age median (IQR)	Clinical outcome, n (%)		
				Active care	Lost to follow-up	Deceased preoperatively
Total (Haiti)	1817	52	5.87 (2.16, 12.31)	1043 (57.4)	529 (29.1)	245 (13.5)
Metro West	828	50	4.62 (1.95, 10.75)	511 (61.7)	202 (24.4)	115 (13.9)
Rest West	467	55	6.18 (2.07, 13.47)	267 (57.2)	141 (30.2)	59 (12.6)
Southeast	47	49	9.25 (3.49, 14.03)	29 (61.7)	11 (23.4)	7 (14.9)
North	49	61	8.83 (5.93. 12.99)	28 (57.1)	17 (34.7)	4 (8.2)
Northeast	17	71	11.33 (5.29, 15.38)	9 (52.9)	5 (29.4)	3 (17.6)
Artibonite	128	45	6.78 (2.99, 11.62)	65 (50.8)	45 (35.2)	18 (14.1)
Center	124	55	8.6 (4.34, 14.74)	42 (33.9)	59 (47.6)	23 (18.5)
South	76	55	7.22 (2.09, 10.21)	46 (60.5)	23 (30.3)	7 (9.2)
Grande d'Anse	20	60	5.44 (2.11, 9.12)	11 (55.0)	7 (35.0)	2 (10.0)
Northwest	23	52	10.65 (4.03, 17.31)	11 (47.8)	9 (39.1)	3 (13.0)
Nippes	38	42	4.09 (1.98, 17.13)	24 (63.2)	10 (26.3)	4 (10.5)

*F*, Female; *IQR*, Interquartile Range

Relative to the general Haitian population, the West department was overrepresented in the HCA patient base (71% of participants). Proportional representation was achieved in the Nippes and Center departments. In other departments, the HCA patient base was somewhat underrepresented relative to the general population (Table 3).

**Table 3:** Percent of Haiti Cardiac Alliance study population residing in each administrative department, compared with the overall Haitian population

	Haiti cardiac alliance cohort (%)	Haitian population (%)
Total (Haiti)	100.0	100.0
West	71.3	38.9
Southeast	2.6	6.9
North	2.7	9.0
Northeast	0.9	3.4
Artibonite	7.0	15.9
Center	6.8	6.6
South	4.2	6.1
Grande d'Anse	1.1	3.7
Northwest	1.3	6.7
Nippes	2.1	2.7

Table 4 shows the population-level health and sociodemographic variables extracted from EMMUS-VI survey that were used in regression analyses at the level of the administrative department.

**Table 4:** Pertinent Haitian population health and sociodemographic variables by administrative department

Administrative department	Prenatal care (%)	Early child mortality (per 1000)	Child growth retardation (%)	Adult employment (%)	Economic index	Average time to nearest healthcare facility (min)
Metro West	91	66	20	52.4	77.79	35.01
Rest West	88.7	84	23	57.8	46.32	54.31
Southeast	91.1	55	20	56.85	39.42	49.17
North	93.4	33	20	53.45	49.34	48.24
Northeast	94.6	54	21	55.7	46.54	38.31
Artibonite	89.8	51	22	55.85	43.4	54.29
Center	92.8	65	30	55	36.1	46.55
South	87.7	40	22	54.85	41.17	56.9
Grande d’Anse	90.3	28	22	60	31.2	58.92
Northwest	95.6	45	20	51.5	41.36	42.4
Nippes	93.2	72	17	59.1	40.95	52.38

### Univariate regression modeling

As exhibited in Table 5, higher departmental rates of childhood growth retardation were associated with: 1) a lower proportion of patients in the *active care* group (OR = 0.979, *p* = 0.002); and 2) a higher proportion of patients in the *lost to follow-up* group (OR = 1.016, *p* = 0.009). All other univariate analyses were statistically non-significant.

**Table 5:** Results of univariate regression analysis predicting Haiti Cardiac Alliance clinical outcomes by administrative department based on population health and sociodemographic variables

	Odds ratio	95% Confidence interval		*p*-value
		Lower bound	Upper bound	
*Prenatal care*				
Active care	0.989	0.968	1.010	0.31
Lost to follow-up	1.008	0.990	1.026	0.43
Deceased preoperatively	1.004	0.996	1.012	0.38
*Early child mortality*				
Active care	1.000	0.997	1.003	0.97
Lost to follow-up	0.999	0.996	1.002	0.51
Deceased preoperatively	1.001	1.000	1.002	0.18
*Child growth retardation*				
Active care	0.979	0.969	0.989	0.002***
Lost to follow-up	1.016	1.006	1.026	0.009***
Deceased preoperatively	1.005	1.000	1.011	0.104
*Adult employment*				
Active care	1.007	0.987	1.028	0.49
Lost to follow-up	0.994	0.978	1.011	0.53
Deceased preoperatively	0.998	0.990	1.006	0.68
*Economic index*				
Active care	1.002	0.998	1.007	0.29
Lost to follow-up	0.997	0.994	1.001	0.17
Deceased preoperatively	1.000	0.998	1.002	0.89
*Time to nearest healthcare facility*				
Active care	1.001	0.994	1.009	0.70
Lost to follow-up	1.001	0.995	1.007	0.79
Deceased preoperatively	0.998	0.995	1.000	0.09*

*p < 0.1, ***p < 0.01

### Multivariable regression modeling

Table 6 displays the results of multivariable, logistic regression modeling of the HCA clinical outcome groups (active care, lost to follow-up, deceased preoperatively) in relation to the selected EMMUS-VI sociodemographic variables, all of which were measured at the departmental level.

**Table 6:** Results of multivariable regression analysis predicting Haiti Cardiac Alliance clinical outcomes by administrative department, based on population health and sociodemographic variables at the department level

Outcome	Odds ratio	95% Confidence interval		*p*-value	Multiple R^2^	F-statistic
		Lower bound	Upper bound			
*Active care*						
Overall model				0.0013***	0.9307	20.14
Prenatal care	0.980	0.971	0.989	0.005***		
Child growth retardation	0.977	0.972	0.983	0.00019****		
Adult employment	1.006	0.996	1.015	0.284		
Time to nearest healthcare facility	0.997	0.993	1.001	0.160		
*Lost to follow-up*						
Overall model				0.005***	0.822	10.77
Prenatal care	1.019	1.008	1.026	0.013**		
Child growth retardation	1.018	1.011	1.025	0.002***		
Time to nearest healthcare facility	1.004	1.000	1.008	0.065*		
*Deceased preoperatively*						
Overall model				0.015**	0.8361	7.653
Prenatal care	0.989	0.981	0.997	0.037**		
Early child mortality	1.001	1.000	1.001	0.085*		
Economic index	0.996	0.995	0.998	0.007***		
Time to nearest healthcare facility	0.992	0.988	0.996	0.0046***		

*p < 0.10, **p < 0.05, ***p < 0.01, ****p < 0.001

Notably, the proportion of department patients in *active care* had a significant, positive association with decreased child growth retardation (OR = 0.977, *p* = 0.00019) and a significant, negative association with access to qualified prenatal care (OR = 0.980, *p* = 0.005). Although not statistically significant, active care status was positively associated with increased adult employment rate (OR = 1.006, *p* = 0.28) and decreased travel time to the nearest healthcare facility in an emergency (OR = 0.997, *p* = 0.16). Overall, this predictive model achieved a multiple R^2^ value of 0.9307 and *p*-value of 0.0013.

Next, the proportion of department patients *lost to follow-up* was significantly, positively associated with: increased child growth retardation (OR = 1.018, *p* = 0.002) and increased access to qualified prenatal care (OR = 1.019, *p* = 0.013). Lost to follow-up status had a nearly significant positive correlation with increased travel time to the nearest healthcare facility in an emergency (OR = 1.004, *p* = 0.065). Overall, this predictive model achieved a multiple R^2^ value of 0.822 and *p*-value of 0.005.

Finally, the proportion of department patients *deceased preoperatively* had a significant, negative association with: access to qualified prenatal care (OR = 0.989, *p* = 0.037), average economic index (OR = 0.996, *p* = 0.0070), and travel time to the nearest health facility in an emergency (OR = 0.992, *p* = 0.0046). Early child mortality had a positive association with *deceased preoperatively* status that was nearly statistically significant (OR = 1.001, *p* = 0.085). Overall, this predictive model achieved a multiple R^2^ value of 0.8361 and *p*-value of 0.015.

## Discussion

Overall, this study of cardiac care in Haiti demonstrates significant associations between ecological independent variables and clinical outcomes. The young median age in the patient database (5.87 years) supports existing evidence that cardiac diseases of childhood, including CHD and RHD, are a notable source of CVD burden in Haiti [6–9, 22]. However, because significant CHD and RHD are often symptomatic, they may rise to clinical attention earlier and more frequently than hypertensive and atherosclerotic forms of CVD. By natural course, hypertension and ASCVD remain asymptomatic for decades; thus, for identification they require routine population-level screening that is largely unavailable in Haiti [23]. The lack of ASCVD patients in the HCA database is unsurprising given the limitations of the primary healthcare system in Haiti to detect these pathologies. Therefore, this study does not provide insight into the comparative burden of hypertension and ASCVD in Haiti relative to CHD and RHD.

These data further revealed that the *West* department was overrepresented in the HCA patient database relative to the population share of *West* in Haiti at large. Meanwhile, the more rural *South, South-East, North, North-East, Grande d’Anse, *and *Artibonite* departments were relatively underrepresented. Beyond the fact that three of the five clinical sites are located in *West,* this patient distribution likely reflects differences in referral patterns due to a higher degree of urbanization in *West *[13], which contains the entire Port-au-Prince metropolitan area. Urbanization may increase HCA referrals because patients are closer to the referring facilities where their cases can be identified and closer to HCA clinics where referrals can be completed. Also, urban patients are more likely to have higher socioeconomic status, which may facilitate care access.

Indeed, the relative paucity of rural patients in the HCA database result aligns with existing literature on rural access barriers to specialty care in low- and middle- income countries. For instance, a 2021 study in rural Madagascar found that even in the presence of referral programs strengthened by the health system, geographic barriers leading to increased referral travel times were a primary driver of diminished access to specialty care in rural Madagascar [24]. Of note, a large portion of HCA patients present for cardiac complications of RHD, which begins with an infectious etiology and is less likely to be adequately treated and prevented in rural settings. Overall, these findings suggest that more robust, decentralized networks of HCA referrals and evaluations are needed to bring cardiac care closer to the rural poor, as exemplified by the World Health Organization PEN-Plus strategy [25].

While most reflective of a similar rural–urban access disparity within Haiti, the urban-heavy distribution of HCA patients may also be compatible with other epidemiologic trends. In a 2018 review, Bickler et al. note that in Sub-Saharan Africa, rates of non-communicable diseases like CHD often rise fastest in the urban regions of a country, as compared to rural and less industrialized areas; this may be partially attributable to documented genetic and epigenetic modifications that accompany urbanization [26], although no research to date explores similar biomolecular changes in a Haitian context.

Additionally, it is critical to note that the sociopolitical circumstances in Haiti during the timeframe of HCA data collection (April 2012–December 2020) may have masked some intra-departmental disparities in access to care. Due to the COVID-19 pandemic and the Threat Level 4 travel advisory issued by the US State Department in response to kidnapping and uprising threats in Haiti, its national border was effectively closed for large parts of 2020 [14]. While HCA continued to operate medically, this translated into a universal halt in surgical therapy (which, due to these security concerns, is now exclusively conducted at HCA’s international partner sites given the absence of cardiac surgical capability in Haiti) because eligible patients could not be transited abroad. This uniform reduction in treatment availability may have diminished some of the regional differences normally present in HCA care outcomes.

Furthermore, patient movement across administrative departments over time was not a significant analytical concern during the time frame of this study; while some patients emigrate abroad after coming into contact with HCA, this also comprised only a small minority of the analyzed dataset. After the conclusion of the study period, however, Haitian population movement increased substantially due to a catastrophic earthquake and widespread sociopolitical unrest following the assassination of former president Jovenel Moïse in 2021. Therefore, future studies of Haitian and LMIC cohorts should also consider the potential of ongoing internal displacement and international migration flows to impact clinical outcomes of the studied population.

Our univariate logistic regression analysis showed a negative association between departmental childhood growth retardation and *active care* representation in HCA. Conversely, higher rates of childhood growth retardation were positively associated with loss to follow-up in HCA. Given the high prevalence of severe malnutrition in Haiti, childhood growth retardation has been identified as a key public health target [27]. In addition, the association between growth retardation and the percentage of patients receiving any form of healthcare is supported by existing literature. Analyses of Haitian national survey data in the aftermath of the 2010 earthquake suggested that rates of under-nutrition among children below five years declined in tandem with increased antenatal care attendance and associated “baby tents” designed to promote infant health [28].

At the department level, multivariable regression models trended towards a positive association of *active care* representation with adult employment rate (although this association was not statistically significant in our sample). Moreover, there was a statistically significant, negative correlation between departmental economic index and rates of preoperative death in HCA. The relationship of adult employment and income to healthcare access is well-established in LMICs given the increased ability of employed individuals to afford out-of-pocket expenses in areas without widespread health insurance [18].

In multivariable models, travel time to the nearest healthcare facility also trended towards a negative association with the proportion of patients in *active care* without reaching the threshold of statistical significance. Similarly, there was a nearly-significant, positive correlation between travel time to emergency healthcare and the departmental rate of HCA patients *lost to follow-up*. Because increased travel time is often interlinked with other social determinants of health such as financial barriers and the cultural acceptability of seeking professional medical services, multivariable analysis here was necessary to parse the individualized contribution of travel time [29]. Travel time has also been identified as a driver of healthcare disparities between urban and rural areas in LMICs [30].

In this study, however, it is worth considering that referral biases may cause overrepresentation of patients with shorter travel times to HCA clinics from within the broader departmental population. For instance, we found that the rate of HCA patients *deceased preoperatively* by department was negatively correlated with travel time to emergency healthcare facilities in multivariable regression. While this unexpected finding requires further investigation, it raises the possibility that a greater portion of symptomatic patients—like those with decompensated CHD or advanced RHD—in departments with accessible, well-dispersed healthcare facilities are more likely to be referred to HCA for advanced disease, whereas symptomatic patients in departments where healthcare facilities are less readily accessible to all may be more likely to die before even making contact with a specialty cardiac provider like HCA.

Notably, the effect sizes for each covariate within these multivariable regressions appear small, as may be reasonably expected when correlating population-level indicators with the clinical outcomes of a specialty cardiac patient group. Importantly, however, the collective set of covariates in each model provides adequate predictive power, as indicated by high R^2^ values. This finding, in turn, demonstrates how aggregate, widely available population health and sociodemographic data predicts clinical outcomes disparities within the particular cardiac patient cohort of Haiti Cardiac Alliance. For this reason, our study suggests that readily available population-level data may be a useful starting point for identifying healthcare inequities confronted by specific public health interventions and policies, specifically within Haiti and possibly in other resource-constrained, data-limited settings as well.

Counterintuitively, *active care* representation was negatively associated with access to qualified prenatal care and positively associated with *lost to follow-up* representation when controlling for other covariates. While a precise mechanism for this finding is unclear, departments with greater availability of qualified prenatal care are also more likely to have other established primary and secondary healthcare providers. It is plausible that patients in these areas may view HCA as a pathway to cardiac surgery rather than as a long-term cardiac care provider, and may transfer their postoperative follow-up care and long-term medical management from HCA to local providers.

This hypothesis is supported by the intuitive finding that prenatal care access was negatively correlated with pre-operative death rates. In other words, the prenatal care variable in EMMUS-VI may serve as an indicator of overall healthcare availability. Departments with weaker regional health systems would presumably have higher preoperative mortality in HCA due to both: a) greater risk of patients facing other unmet health needs and/or medical emergencies while awaiting cardiac surgery; and b) delayed referral to secondary healthcare services like HCA, until surgery is no longer clinically feasible and mortality is imminent. At the same time, departments with stronger regional health systems might plausibly be less dependent on HCA for long-term medical management of postoperative patients, who may transfer their care to regional providers postoperatively and lose contact with HCA over time.

### Strengths, limitations & future directions

This study has numerous strengths. It is the first analysis of Haiti’s largest known cardiac patient database, which has significant statistical power owing to a large sample size. This rare source of patient-based data from Haiti allows us to offer one of the first academic approaches to characterizing regional health disparities within Haiti, as defined by clinical outcomes. Because the study uses data derived from zero-cost cardiac consultations in public and aid-based clinics, it is broadly inclusive and likely to represent the general Haitian population with a high degree of external validity. Drawing from multiple clinic sites across Haiti, the study includes data from all 10 administrative departments and thus provides a national-level picture of the cardiac care landscape.

As opposed to current literature that primarily describes healthcare access in specific settings of Haiti [17, 22], this study is also among the first to identify factors associated with current disparities in cardiac care access using readily available department-level sociodemographic data. Using objective data from the EMMUS-VI national survey, the study proposes a starting point to triangulate “access to care,” a concept that is difficult to measure directly. This difficulty is particularly pronounced in Haiti, where experiences of poverty and resource scarcity are quite heterogeneous between rural and urban settings. In these ways, our study draws from department-level data on both HCA clinical outcomes and national sociodemographic and population health indices to advance the literature on social determinants of health in Haiti.

This study is not without limitations, which were often imposed by data availability. First, defining clinical outcomes in three broad categories (*active care, deceased preoperatively,* or *lost to follow-up)* does not account for the full spectrum of clinical experiences over time. For instance, departments in which a high proportion of patients have been in consistent active care for years may differ from those where most patients have been in active care for weeks to months, yet our classification system does not reflect these differences. It also does not differentiate patients with sustained active care from those who were lost to follow-up for some time and subsequently returned to care (possibly after interim disease progression). Moreover, while risks of patient misclassification are not uncommon in data analyses given the possibility of primary data source errors, Haiti Cardiac Alliance mitigates these risks through fieldwork protocols that involve frequent updates and routine quality controls.

Second, we were unable to analyze patients by type or severity of cardiac pathology, which may substantially impact clinical outcomes. For example, medically-treated patients for whom surgery is recommended are especially vulnerable because they face higher mortality rates without surgical intervention [31, 32], despite optimal medical management; our study merges this group with patients who lack operative indications. On the other hand, many patients with surgical cardiac pathologies present to HCA at such an advanced stage in the natural course of their disease that surgery is prohibitively high-risk. Because we could not explicitly label or know if patients' primary reason for death was structural unavailability of surgery, there is potential for lead-time bias as patients screened or diagnosed early on in a severe or unalterable course of disease would be classified in the *active care* group as opposed to the *deceased preoperatively* group. Nevertheless, it is important to note that this study does not serve to evaluate effectiveness of HCA operations or therapeutic interventions, but rather baseline care access across Haiti. Therefore, we believe that including mortalities during treatment in the *active care* group still provides a reliable marker of patients’ access to care, in line with the study aims. As such, we do not believe these biases would have negated the impact shown by our statistical analysis of departmental sociodemographic factors given the opportunities to access and remain in care during our project timeframe.

Relatedly, the ability to segment patients by more granular characteristics of clinical status or disease could have improved interpretation by allowing for sensitivity analyses and quality controls in which illness severity could have been incorporated as a covariate. While disparities in the types of cardiac pathologies seen across different administrative departments of Haiti would translate to interregional differences in clinical outcome status, the current analyses are based on the underlying assumption that different pathologies are approximately evenly distributed across Haiti’s departments. RHD is a post-infectious condition where prevalence may vary based on the robustness of public health infrastructure in different regions, but overall, this assumption of homogeneity is a reasonable approximation given that a large share of the observed presentations is secondary to spontaneous congenital anomalies.

Third, the HCA database includes only patients for whom information about treatment outcome and departmental location was available; excluded patients might have altered aggregate outcomes in meaningful ways. Finally, given the lack of available sociodemographic data at finer administrative levels like the commune or section, our analysis cannot address cardiac care access barriers within a given department.

Importantly, all findings of this study should be interpreted as hypothesis-generating. Future studies should aim to model cardiac care disparities in Haiti with greater precision by using patient-level data (home address, income or other indicators of economic status, travel time to cardiac clinic, employment status, surgical vs non-surgical cardiac pathology, and comorbidities) to predict clinical patient outcomes. To increase internal validity and better inform policymaking, regional-level studies of HCA data should be conducted at the neighborhood level. In this way, precise estimates of on-the-ground travel time and travel distance to the nearest cardiac clinic may be incorporated.

## Conclusions

In conclusion, this study is the first to model cardiac clinical outcomes in Haiti based on population health and sociodemographic indices at the level of the administrative department. By identifying the departments most underrepresented in the HCA cohort as well as those with suboptimal clinical outcomes, this study may inform future public health interventions and policies designed to mitigate geographic disparities in cardiac care access and enhance health equity across Haiti. Methodologically, our work serves as a starting point for better understanding regional health disparities within LMICs through analysis of widely available, population-level health and sociodemographic data. Therefore, these data may contribute to the development of practical, generalizable models for health outcomes analysis in underserved settings with minimal data availability. In summary, our study offers novel insights into regional disparities in cardiac care access across Haiti, which may have implications for the prevention and treatment of noncommunicable diseases in LMICs more broadly.

## Data Availability

The *2016–2017 Enquête Mortalité, Morbidité et Utilisation des Services* (EMMUS-VI) *[Study of mortality, morbidity and utilisation of services]* dataset analysed during the current study is available in the Demographic and Health Surveys (DHS) Program of the United States Agency for International Development (USAID) repository, https://www.dhsprogram.com/pubs/pdf/FR326/FR326.pdf. The Haiti Cardiac Alliance dataset analysed during the current study is not publicly available due to organizational policy, but is available from the corresponding author on reasonable request.

## Abbreviations

HCA: Haiti cardiac alliance
CVD: Cardiovascular disease
ASCVD: Atherosclerotic cardiovascular disease
CHD: Congenital heart disease
RHD: Rheumatic heart disease
EMMUS-VI: Enquête Mortalité, Morbidité et Utilisation des Services, 2016-2017 [Study of mortality, morbidity and utilisation of services]
LMICs: Low- and middle- income countries
CIs: Confidence intervals
VIF: Variance-inflation factor
F: Female
IQR: Interquartile range
